# Comparison of pregnancy outcomes after history-indicated and ultrasound-indicated cervical cerclage: A systematic review and meta-analysis

**DOI:** 10.1371/journal.pone.0328564

**Published:** 2025-08-14

**Authors:** Jiamei Wang, Ling Zhu, Chunyan Xu, Wenjun Wu, Xuya Shen

**Affiliations:** 1 Nursing Department, Huzhou Maternity & Child Health Care Hospital, Huzhou City, Zhejiang Province, China; 2 B-ultrasound Room, Huzhou Maternity & Child Health Care Hospital, Huzhou City, Zhejiang Province, China; 3 Obstetrical Department, Huzhou Maternity&Child Health Care Hospital, Huzhou City, Zhejiang Province, China; Athens Medical Group, Psychiko Clinic, GREECE

## Abstract

**Objective:**

To compare maternal and neonatal outcomes in women with a previous history of pregnancy loss and/or preterm delivery who underwent ultrasound-indicated cerclage (UIC) or history-indicated cerclage (HIC).

**Methods:**

PubMed, Web of Science, Scopus, and Embase databases were searched for observational studies and randomized controlled trials (RCT) from inception to 30 April 2024. Eligible studies should have compared the outcomes of women with singleton pregnancies who underwent UIC or HIC. STATA version 15.0 was employed, and the analysis was done using a random effects model and unadjusted effect sizes from the included studies.

**Results:**

Of 25 included studies (n = 3909), most (n = 18) were retrospective cohort studies. Compared to women who underwent HIC, UIC was associated with higher risk of having a preterm birth (<37 weeks of gestation) (OR 1.48, 95% CI: 1.17, 1.88; N = 15), low birth weight (<2500g) (OR 1.78, 95% CI: 1.32, 2.41; N = 6) and admission to neonatal intensive care unit (OR 1.70, 95% CI: 1.27, 2.27; N = 6,). Women with UIC also had a higher risk of chorioamnionitis (OR 2.34, 95% CI: 1.36, 4.04; N = 4). The risk of having a low APGAR score (5-minute score of less than 7), fetal death and preterm premature rupture of membrane (PPROM) was comparable among the two groups.

**Conclusion:**

Our results demonstrate that UIC is associated with higher risks of adverse pregnancy outcomes compared to HIC. However, our evidence emanates from observational studies and is prone to biases, particularly because the findings were unadjusted for potential confounders. More clinical trials are needed to confirm our observations.

**Systematic review protocol registration:**

PROSPERO CRD42024544181

## Introduction

Cervical cerclage is a surgical intervention commonly used to prevent preterm birth in women at high risk of cervical insufficiency [1,2]. This procedure involves the placement of sutures around the cervix to reinforce it and prevent premature dilation. Two primary indications for cervical cerclage are history-indicated cerclage (HIC), based on a patient’s obstetric history, and ultrasound-indicated cerclage (UIC), determined by ultrasonographic evidence of cervical shortening during pregnancy [2]. While cervical cerclage has been shown to reduce the risk of preterm birth and perinatal mortality efficiently [3], the decision to perform a procedure based on historical factors versus ultrasound findings remains a topic of considerable clinical interest and debate [4]. HIC is typically recommended for women with a history of second-trimester losses or preterm births attributed to painless cervical dilation [2,5]. Conversely, UIC is performed when a significant shortening of the cervix is observed via transvaginal ultrasound, often identified during routine mid-trimester scans [2,5].

Given the differences in indications and timing for these two approaches, it is essential to evaluate and compare their effectiveness in improving pregnancy outcomes. A previous review by Berghella et al.[6] compared pregnancy outcomes of women with singleton pregnancies and a history of preterm birth that had either cerclage placed after cervical length screening or HIC. The review included 4 randomized trials (n = 476 subjects) and showed both approaches were associated with comparable rates of preterm birth and perinatal mortality [6]. The review concluded that singleton pregnancies in women with a history of preterm birth can be safely monitored with transvaginal ultrasound cervical length screening rather than routine HIC. However, the review included small number of studies, focused on limited outcomes and did not take into account non-randomized studies that utilized cohort-based data [6]. Another systematic review by Blikman et al included 6 studies and compared pregnancy outcomes in patients at risk of cervical insufficiency who received either UIC or HIC [7]. Five out of 6 included studies did not report differences in pregnancy outcomes (preterm birth or pregnancy loss <24 weeks) between the two groups. However, as new studies on the subject have been conducted since the publication of these reviews, there is a need for an updated review of contemporary data.

This study aims to conduct an updated systematic review of the literature comparing HIC and UIC with the aim to provide critical insights into the relative benefits of each approach, thereby guiding clinical practice and decision-making. We focused on outcomes that were not necessarily restricted to preterm birth but extended to other foetal and maternal outcomes.

## Methodological procedures

### Article search and screening criteria

PubMed, Web of Science, Scopus, and Embase databases were screened. Studies published until 30^th^ April 2024 were eligible for consideration. Specific keywords were used: (“pregnancy outcomes” OR “maternal-fetal outcomes” OR “perinatal outcomes” OR “preterm labor” OR “premature birth” OR “obstetric complications”) AND (“history-indicated cervical cerclage” OR “ultrasound-indicated cervical cerclage” OR “ultrasound-guided cervical cerclage”) AND (“cervical incompetence” OR “cervical insufficiency”). The PRISMA guidelines were followed [8].

Population (P): Pregnant individuals with singleton pregnancies and a documented indication for cervical cerclage, based either on historical obstetric data or ultrasonographic findings.

Intervention (I): History-Indicated Cerclage (HIC) – placement of cerclage based on prior obstetric history.

Comparison (C): Ultrasound-Indicated Cerclage (UIC) – placement of cerclage based on ultrasonographic evidence, such as cervical shortening.

Outcomes (O): At least one of the following pregnancy outcomes:

- Preterm birth
- Gestational age at delivery
- Foetal/perinatal mortality
- Birth weight
- Other relevant maternal and neonatal outcomes

### Study design

Randomized controlled trials (RCTs) and observational studies (e.g., cohort and case-control studies) were eligible. Studies with either adjusted or unadjusted effect sizes were included. We included both RCTs and observational studies to ensure a comprehensive synthesis of the available evidence, as the clinical nature of cervical cerclage and ethical considerations often limit the feasibility of conducting RCTs for all subtypes. Observational studies provide valuable real-world data on the effectiveness and safety of cerclage types across diverse clinical populations. Including both study designs allowed for a broader and more representative understanding of the comparative outcomes associated with HIC and UIC.

Excluded: Studies solely focussing on one type of cerclage, without comparing it to the other intervention, those involving multiple gestations (e.g., twins, triplets), and those lacking sufficient outcome data or methodological rigor, such as case reports, letters, editorials, and conference abstracts.

No restrictions were placed on publication language or geographical location

### Selection of studies, their data extraction and quality assessment

Studies identified by the literature search were deduplicated. Titles and abstracts of the remaining studies were scrutinized to identify those potentially relevant to the research objective. Subsequently, full texts of these potentially relevant studies were reviewed, leading to further exclusions and resulting in the final selection of studies for inclusion in the review process. Two authors (JW, LZ) independently carried out each step of this selection process to ensure reliability and accuracy.

Two authors (CX, WW) independently extracted relevant data from the final set of studies using a structured extraction form. This form was developed collaboratively through mutual discussion among the authors. The information extracted from the eligible studies was related to study identifier (author and year of publication), design, place of conduct, subject characteristics, gestational age at the time of cerclage, sample size, cervical length cut-off used for UIC, outcomes presented in the study and key findings. The quality of observational studies was assessed using the Newcastle-Ottawa Scale (NOS) [9]. For randomized controlled trials (RCTs), the Cochrane Risk of Bias (RoB 2.0) tool was used [10]. Any discrepancies or disagreements that arose during these processes were resolved through discussions.

### Statistical analysis

STATA version 15.0 was used for analyses. We did not perform any imputation for missing data. Our analysis was based solely on the data as reported in the included studies. Wherever information was unavailable or not reported, those data points were excluded from the relevant analyses. Effect sizes, including odds ratios (OR) for categorical outcomes or weighted mean differences (WMD) for continuous outcomes along with their respective 95% confidence intervals (CI), were computed utilizing a random-effects model. This approach was adopted to accommodate potential variations in participant characteristics and methodological differences across the studies included in the analysis [11]. Separate analyses were conducted for observational studies and RCTs. Publication bias was assessed by funnel plots and Egger’s test [12]. P < 0.05 was considered significant.

### Results

Database search identified 338 studies (S1 Fig). Of them, 55 duplicate records were removed, and the remaining 283 studies underwent screening based on their titles and abstracts. After this screening phase, 249 studies were excluded. The full texts of the remaining 34 studies were reviewed. Finally, 25 studies met the predetermined eligibility criteria and were included in the meta-analysis (S1 Fig) [13–37]. Studies contributed to an overall sample of 3909. Most studies were retrospective cohort (n = 18) (Table 1). Additionally, four studies were RCTs, two were case-control studies and one was a prospective cohort study. Most studies were from the USA (n = 8) and the United Kingdom (n = 5), followed by two studies each from Japan and Turkey. In 20 studies, the cervical length cut-off for UIC was set at <25mm (Table 1). The included cohort studies were of acceptable quality, as indicated by the mean NOS score of 7.1. Quality assessment of the 4 RCTs is presented as S2 Fig. Almost all studies either presented the non-confounder adjusted effect sizes or we had to derive the unadjusted effect sizes based on the data provided within the individual studies.

**Table 1:** Studies included and their characteristics.

Author	Study design; location	Population	Gestational age at time of cerclage	Cervical length Cutoff (mm) for cerclage	Sample size	NOS Quality score
Ikechebelu et al (2023)	RC; Nigeria	Women with singleton pregnancy who underwent cerclage; no significant difference between the two groups in terms of maternal age, parity and body mass index	HIC: mean of 14.4 wks UIC- mean of 14.7 wks	<25 mm	103 (HIC: 68; UIC: 35)	7
Mullin et al (2023)	RC; United Kingdom	Women with singleton pregnancy who underwent transvaginal cerclage; similar age (mean of 34 years) and BMI in both groups; higher proportion with a prior mid-trimester pregnancy loss in the HIC group while there was a higher proportion with a prior PT birth in UIC group	HIC: mean 14.9 wks UIC- mean 18.3 wks	<25mm	189 (HIC: 66; UIC: 123)	6
Adekola et al (2022)	RC; USA	Women with singleton pregnancy who underwent transvaginal cerclage; similar age (mean 31 years), gravidity and tobacco use in both groups; higher proportion with previous PT birth in HIC group	HIC: mean 20.5 wks UIC- mean 15.0 wks	<25 mm	72 (HIC: 39; UIC: 33)	6
Seyama et al (2022)	RC; Japan	Women aged ≥20 years, with history of PT birth and with viable singleton pregnancy who received cervical cerclage; mean age similar in both groups (33 years); BMI/ Parity/history of PT birth similar in both groups	HIC: 12–14 wks UIC: < 28 wks	<25 mm	444 (HIC: 388; UIC: 56)	7
Golbasi et al (2022)	RC; Turkey	Women who underwent cerclage with the diagnosis of cervical insufficiency; no significant difference between groups in terms of maternal age (mean age 30 years), parity, miscarriage, and maternal smoking	HIC: mean 16.1 wks UIC: mean 18.1 wks	<25 mm	58 (HIC: 41; UIC: 17)	7
Kuruma et al (2022)	RC; Japan	Women with singleton pregnancy with cerclage; similar age (mean of 34 years), BMI and parity in both groups; higher proportion with a prior 2nd trimester pregnancy loss and PT birth in HIC group	HIC: mean 13.6 wks UIC- mean 21.3 wks	<25mm	134 (HIC: 38; UIC: 96)	7
Yüksel et al (2021)	RC; Turkey	Women with singleton pregnancy with cerclage; similar age (mean of 30 years); higher BMI in UIC group	HIC: mean 13.9 wks UIC- mean 19.4 wks	<25mm	75 (HIC: 48; UIC: 27)	7
Chen et al (2020)	RC; China	Women with viable singleton pregnancy who received cervical cerclage; mean age similar in both groups (31 years); BMI/gravidity/Parity/history of PT birth and second trimester pregnancy loss similar in both groups	Median for HIC (15.7 wks) Median for UIC (19.1 wks)	<25 mm	291 (HIC: 232; UIC: 59)	8
Battarbee et al (2019)	RC; USA	Women with a singleton, non-anomalous foetus with cerclage in situ; Mean age 32 years, mean BMI of 29 kg/m2; more than 75% with previous spontaneous PT birth	Mean GA at cerclage placement 14.3 wks	<25mm	79 (HIC: 59; UIC: 20)	7
Story et al (2017)	CC; United Kingdom	Women with singleton pregnancy who underwent cerclage; similar age, BMI and parity in both groups	HIC: 12–14 wks UIC- 14–24 wks	Not provided	119 (HIC: 68; UIC: 51)	6
Suhag et al (2016)	RC; USA	Women with singleton pregnancy who received cervical cerclage; median age lower in UIC group (27 vs. 30 years); similar gravidity and parity in both group; Prior PT birth and second trimester pregnancy loss higher in HIC	HIC: mean of 13.3 wks UIC- mean of 20 wks	<25 mm	375 (HIC: 177; UIC: 198)	8
Chan et al (2015)	RC; Hong Kong	Women with singleton pregnancy who underwent cerclage; no significant difference between the two groups in terms of maternal age (mean 35 years), BMI, parity, prior PT birth and second trimester pregnancy loss	HIC: mean of 14.6 wks UIC- mean of 18.6 wks	<25 mm	38 (HIC: 23; UIC: 15)	7
Kuon et al (2015)	RC; Germany	Women with mostly singleton pregnancy who underwent cerclage; similar age (mean of 32 years) and gravidity in both groups; higher proportion with a prior 2^nd^ trimester pregnancy loss in the HIC group; similar proportion with a prior PT birth in both groups	HIC: mean 19.0 wks UIC- mean 15.0 wks	<25mm	90 (HIC: 48; UIC: 42)	8
Giraldo-Isaza et al (2013)	RC; USA	Women with singleton pregnancy with transvaginal cerclage; similar age (mean 30 years), gravidity and parity in both groups; higher prior 2nd trimester pregnancy loss and PT birth in HIC group	HIC: mean 14.4 wks UIC- mean 19.48 wks	<25mm	444 (HIC: 237; UIC: 207)	8
Drassinower et al (2011)	RC; USA	Women with singleton pregnancy who underwent cerclage; similar age (mean 33 years), BMI and prior PT birth and second trimester pregnancy loss in both groups; higher proportion with multiparity in the UIC group	HIC: mean of 13.6 wks UIC- mean of 19.6 wks	<25 mm	287 (HIC: 198; UIC: 89)	8
Nelson et al (2009)	RC; USA	Women with singleton pregnancy who underwent cerclage with the diagnosis of cervical insufficiency; no significant difference between groups in terms of maternal age (mean age 25 years), gravidity and parity; history of previous PT birth higher in HIC group	HIC: mean of 14.5 wks UIC- mean of 20.3 wks	<25 mm	115 (HIC: 89; UIC: 26)	8
Simcox et al (2009)	RCT; United Kingdom	Pregnant women with singleton pregnancy with at least 1 previous spontaneous delivery between 16 to and 34 weeks of gestation.	HIC: mean 15 wks UIC- mean 19.3 wks	<20 mm	248 (HIC: 125; UIC: 123)	---
Beigi et al (2005)	RCT; Iran	Women with singleton pregnancy and with an obstetric history of spontaneous mid-trimester loss or early preterm delivery; no significant differences in age, BMI, smoking, parity among groups	HIC: mean 14.2 wks UIC- mean 20.1 wks	<20 mm	73 (HIC: 45; UIC: 28)	---
Groom et al (2004)	CC; United Kingdom	Women with a past history of preterm delivery, second trimester loss and with current singleton pregnancy; similar maternal age, history of first trimester pregnancy terminations, and number of second trimester losses and early preterm deliveries among both groups	HIC: mean 13.0 wks UIC- mean 17.4 wks	<25mm	78 (HIC: 39; UIC: 39)	7
Higgins et al (2004)	PC; Australia	Patients at risk of cervical incompetence or early preterm delivery with singleton gestations; similar age (mean 32 years), prior 2nd trimester pregnancy loss and PT birth in both group	HIC: 12–20 wks UIC: ≤ 24 wks	<25mm	135 (HIC: 97; UIC: 38)	6
Berghella et al (2002)	RC; USA	Women with singleton pregnancy; similar age (mean of 30 years), parity and proportion smokers	HIC: 12–15 wks UIC: < 24 wks	<25mm	177 (HIC: 66; UIC: 111)	7
To MS et al (2002)	RC; United Kingdom	Those with singleton pregnancies and a history of one or more spontaneous losses between 16 and 33 weeks. Groups not significantly different BMI, maternal age or incidence of smoking	HIC: mean 13.5 wks UIC- mean 17.6 wks	<25mm	67 (HIC: 41; UIC: 26)	7
Kassanos et al (2001)	RCT; Greece	Patients with a history of previous (one or more) mid-trimester miscarriage and currently having singleton pregnancy	HIC: 14 wks UIC: Not reported	≤20 mm	45 (HIC: 18; UIC: 27)	---
Kelly et al (2001)	RC; USA	Women with singleton pregnancy and with history of spontaneous mid-trimester loss or early preterm delivery; no significant differences in age, gravidity and prior 2nd trimester pregnancy loss among groups	HIC: mean 12.7 wks UIC- mean 17.8 wks	Physician preference	106 (HIC: 45; UIC: 61)	7
Althuisius et al (2000)	RCT; Netherlands	Women with singleton pregnancy and with history of spontaneous preterm delivery or premature rupture of membrane; no significant differences in age, parity, smoking and prior mid-trimester pregnancy loss among groups	HIC: 10–12 wks UIC: 15–27 wks	< 25 mm	67 (HIC: 23; UIC: 44)	---

RC- retrospective cohort; CC- case control; PC- prospective cohort; HIC- history indicated cerclage; UIC- ultrasound indicated cerclage; PT- preterm; BMI- body mass index; RCT- randomized controlled trial; NOS- Newcastle Ottawa Scale Score

### Neonatal outcomes

**Fig 1:**
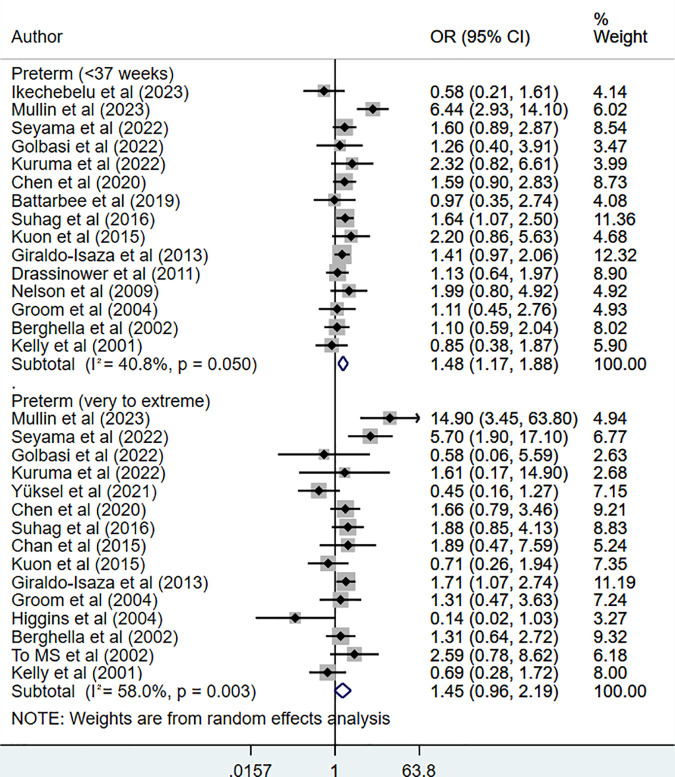
Risk of preterm birth in those undergoing ultrasound-indicated cerclage, compared to history-indicated cerclage.

Compared to HIC, UIC was associated with increased risk of preterm birth (delivery prior to 37 weeks of gestation) (OR 1.48, 95% CI: 1.17, 1.88; N = 15, I^2^ = 40.8%) (Fig 1), and higher risk of having very to extremely preterm birth (gestation under 32 weeks) that approached statistical significance (OR 1.45, 95% CI: 0.96, 2.19; N = 15, I^2^ = 58.0%) (Fig 1). There was no indication of publication bias (Egger’s p-value 0.98) on funnel plot (S3A Fig). When studies with a randomized controlled design were pooled together, pooled estimates showed similar risk of preterm birth in HIC and UIC groups, i.e., < 37 weeks (OR 1.52, 95% CI: 0.65, 3.54; N = 2, I^2^ = 9.6%) and <32 weeks (OR 1.03, 95% CI: 0.60, 1.75; N = 4, I^2^ = 0.0%) (S4 Fig).

**Fig 2:**
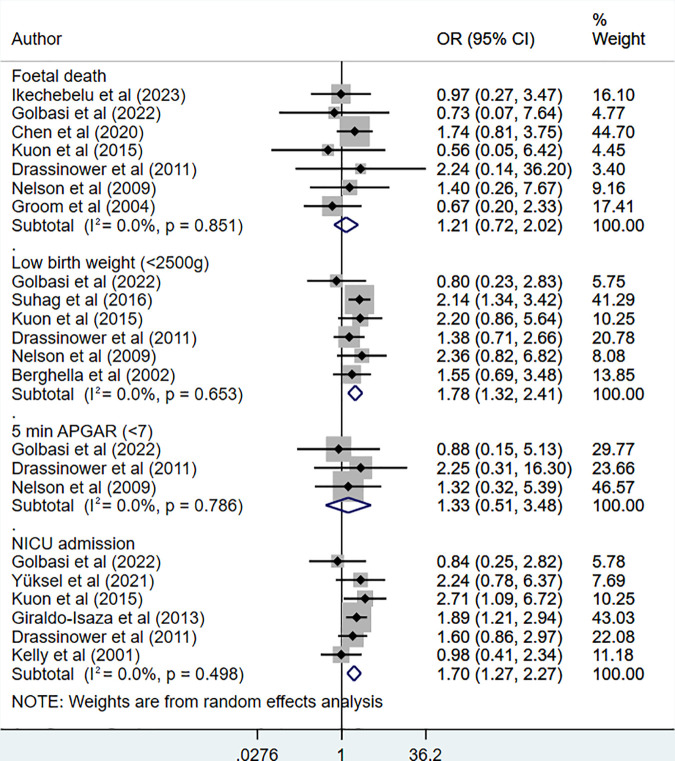
Risk of low birth weight, admission to NICU, low APGAR score and foetal death in those undergoing ultrasound-indicated cerclage, compared to history-indicated cerclage.

UIC was linked to the increased risk of having a child with low birth weight (<2500g) (OR 1.78, 95% CI: 1.32, 2.41; N = 6, I^2^ = 0.0%) and of admission to neonatal intensive care unit (OR 1.70, 95% CI: 1.27, 2.27; N = 6, I^2^ = 0.0%). The risk of having low APGAR score (5-minute score of less than 7) (OR 1.33, 95% CI: 0.51, 3.48; N = 3, I^2^ = 0.0%) and of foetal death (OR 1.21, 95% CI: 0.72, 2.02; N = 7, I^2^ = 0.0%) were comparable between the two groups (Fig 2). In analysis based on RCTs, risk of foetal death was similar in the two groups (OR 0.58, 95% CI: 0.26, 1.27; N = 3, I^2^ = 0.0%) (S4 Fig). There was no indication of publication bias on Egger’s test (p-value 0.38 for low birth weight, 0.54 for admission to NICU, 0.78 for low APGAR score and 0.32 for foetal death) as well on visual inspection of funnel plots (S3B-E Fig).

**Fig 3:**
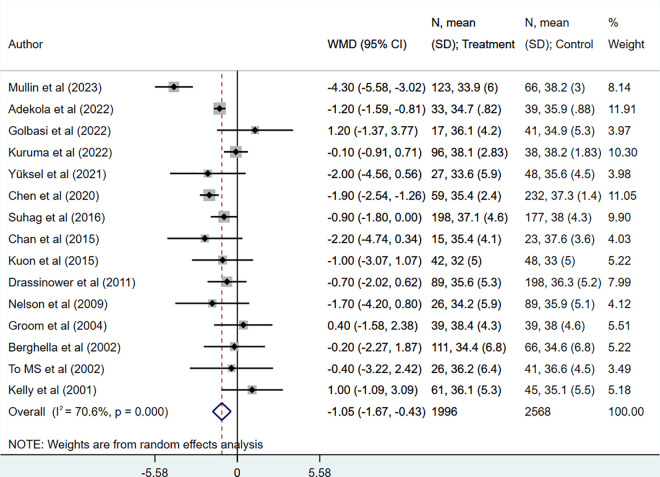
Comparison of gestational age (in weeks) in those undergoing ultrasound-indicated cerclage, compared to history-indicated cerclage.

**Fig 4:**
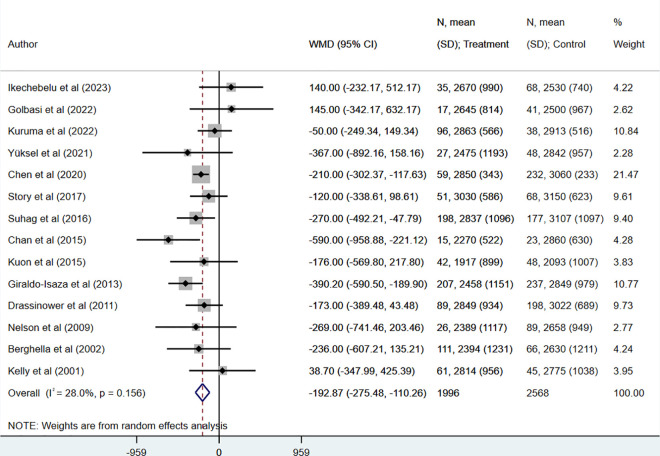
Comparison of birth weight (in grams) in those undergoing ultrasound-indicated cerclage, compared to history-indicated cerclage.

Mean gestational age (in weeks) (WMD −1.05, 95% CI: −1.67, −0.43; N = 15, I^2^ = 70.6%) (Fig 3) and birth weight (in grams) (WMD −192.87, 95% CI: −275.48, −110.26; N = 14, I^2^ = 20.8%) (Fig 4) was also lower in women who underwent UIC, compared to HIC. However, when RCTs were pooled, the mean gestational age was comparable across the two groups (WMD −0.02, 95% CI: −1.49, 1.46; N = 3, I^2^ = 53.8%) (S5 Fig).

### Maternal outcomes

**Fig 5:**
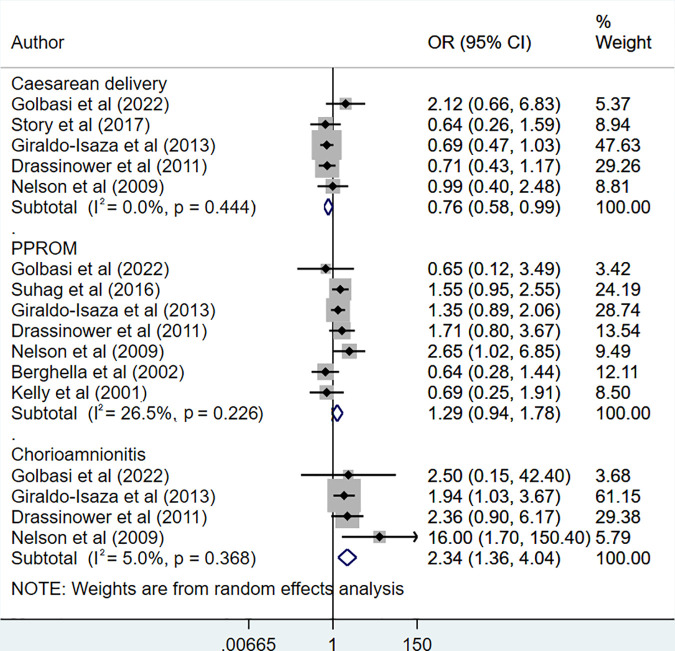
Risk of caesarean delivery, preterm premature rupture of membrane (PPROM) and chorioamnionitis in those undergoing ultrasound-indicated cerclage, compared to history-indicated cerclage.

Women with UIC had increased risk of chorioamnionitis (OR 2.34, 95% CI: 1.36, 4.04; N = 4, I^2^ = 5.0%) and higher, but not statistically significant risk of preterm premature rupture of membrane (PPROM) (Fig 5). UIC was associated with the reduced risk of having a caesarean delivery (OR 0.76, 95% CI: 0.58, 0.99; N = 5, I^2^ = 0.0%) (Fig 5). There was no indication of publication bias on Egger’s test (p-value 0.28 for chorioamnionitis, 0.45 for PPROM, 0.17 for caesarean delivery) as well on visual inspection of funnel plots (S3F-H Fig).

## Discussion

This study compared pregnancy outcomes of HIC and UIC, and showed that UIC was associated with higher risks of adverse pregnancy outcomes compared to HIC. Women undergoing UIC typically present with a shortened cervix during the second trimester, which may reflect an already ongoing subclinical inflammatory or infectious process or cervical insufficiency. This underlying pathology may predispose these pregnancies to preterm birth, chorioamnionitis, and subsequent neonatal complications, regardless of cerclage placement. In contrast, women in the HIC group often receive cerclage earlier in pregnancy based on obstetric history alone, before signs of cervical shortening emerge, potentially preventing cervical changes and exposure to ascending infection. Furthermore, differences in cervical tissue biology at the time of cerclage placement may influence outcomes. By the time UIC is indicated, cervical collagen remodelling and local immune responses may have already advanced, reducing the efficacy of cerclage in preventing further dilation or infection. It is also possible that in UIC, the cervix is already shortened and structurally compromised, making the surgical placement of cerclage technically more challenging and potentially less effective in maintaining cervical competence. In contrast, earlier placement in the HIC group may provide more robust mechanical support throughout gestation. In summary, the differences in baseline risk profiles, timing of intervention, and underlying pathophysiology may contribute to the observed differences in outcomes.

The observed increased risk of preterm birth among women with UIC might underscore the importance of carefully considering indications for cerclage placement. It is important to note that this finding is contrary to the previous review by Blikman et al that found no differences in risk of preterm birth between UIC and HIC [7]. However, the review by Blikman et al. included limited number of studies (n = 6) whereas, the current review summarized data from much larger number of reports. This observed increased risk of preterm births prompts exploration into potential biological mechanisms that may explain the association. One plausible explanation could be related to the selection bias inherent in ultrasound-based indications. Ultrasound screening is able to detect even most subtle cervical changes that may indicate impending preterm labour [38], and timely identify underlying pathological processes such as cervical inflammation or cervical insufficiency. On the other hand, historical indications may capture a broader spectrum of risk factors, potentially leading to more effective risk estimation [39,40]. Further studies of the molecular pathways underlying cervical remodelling and inflammation may shed light on the differential effectiveness of cerclage indications.

Furthermore, the observed association between UIC and higher risk of adverse neonatal outcomes, including low birth weight and admission to the neonatal intensive care unit, highlights the potential clinical implications of cerclage indication and should be confirmed in future studies. Our findings emphasize the need for careful patient selection and counselling regarding risks and benefits of cerclage placement. Interestingly, our analysis revealed a reduced risk of caesarean delivery among women with UIC. While the reasons for this finding are not entirely clear, it might be attributed to differences in cervical length screening protocols, patient characteristics, or obstetric management strategies between the two groups. The observed increased risk of maternal complications, including chorioamnionitis and preterm premature rupture of membranes (PPROM), among women with UIC raises important considerations regarding the potential impact of cerclage indication on maternal health. Our findings underscore the importance of comprehensive maternal risk assessment and close monitoring during pregnancy following cerclage placement.

The lack of significant differences between the two groups when RCTs were pooled could be attributed to several important factors. Firstly, RCTs are considered the gold standard for evaluating interventions, as they minimize bias and confounding variables through randomization. In the case of cerclage indications, RCTs may have implemented strict inclusion criteria and standardized protocols, resulting in less biased outcomes between HIC and UIC groups. Additionally, there is a possibility that due to the small sample sizes of RCTs, the statistical power to detect significant differences between the groups was limited. Further research, including larger RCTs with standardized protocols and long-term follow-up, may help elucidate the differential effects of HIC and UIC on pregnancy outcomes

Clinicians should carefully weigh the risks and benefits of different cerclage indications, considering individual patient characteristics and obstetric history. While ultrasound screening may offer valuable insights into cervical dynamics, close monitoring and multidisciplinary management are essential to mitigate the risks of adverse neonatal and maternal outcomes associated with UIC. Future research should focus on elucidating the underlying biological mechanisms of the observed associations. Prospective studies, preferably clinical trials, with standardized protocols, comprehensive outcome assessments and adequate statistical analysis are needed to provide robust evidence for clinical decision-making.

Our review has several limitations. The most important limitation is the use of non-confounder-adjusted effect sizes from individual studies which may have led to pooled effect sizes that do not reflect the true association. The retrospective nature of studies may have introduced a bias. Additionally, limited number of RCTs included in our analysis may have affected the precision of our estimates. It is important to note that while our review compares outcomes between UIC and HIC, these interventions are applied in distinct clinical contexts: UIC is typically used as an emergency measure based on cervical shortening or funneling, where the interval between cerclage placement and delivery is a key prognostic factor, whereas HIC is a preventive intervention based on obstetric history, with gestational age at delivery being more relevant to assess effectiveness. Therefore, while comparisons provide insights into relative outcomes, the two groups are not directly comparable due to these fundamental differences in indication and timing. Further, although sub-classifying the UIC group based on cervical length thresholds (e.g., < 20 mm, < 15 mm, or open cervix) may have provided more granular insights, this was not feasible due to limitations in the available data. In our review, 20 out of 25 studies used a uniform cervical length cut-off of <25 mm, while only 3 studies used a stricter threshold of <20 mm. The lack of variation in the cut-off used precluded a reliable subgroup analysis. We acknowledge that our search did not include clinical trial registries or grey literature sources. We chose to focus on peer-reviewed, published literature to ensure the inclusion of studies with sufficient methodological rigor and outcome reporting. While we recognize that grey literature and trial registries can reduce publication bias, our priority was to maintain the quality and reliability of data for meta-analysis. In addition, while most outcomes in our analysis showed low heterogeneity (I^2^ < 50%), moderate heterogeneity was noted for very to extreme preterm birth and mean gestational age. This likely reflects differences in gestational age assessment methods across studies, particularly the limited use of early pregnancy ultrasonography in some settings. Variation in study populations and healthcare contexts may also have contributed. Lastly, we did not conduct a GRADE assessment. However, the certainty of evidence is likely to be low, given that most of the included studies were observational in nature and did not adjust for potential confounders.

## Conclusion

Our findings from pooling of non-confounder-adjusted estimates suggest that the mode of indication for cervical cerclage placement may influence pregnancy and maternal outcomes in women with a history of preterm delivery. Future studies should explore reliability and validity of our findings through adoption of robust methodology, including adjustment for important confounders, and also focus on elucidating the underlying mechanisms driving the associations. Ultimately, a personalized approach to cerclage indication and obstetric management tailored to individual patient characteristics and risk profiles may optimize pregnancy outcomes in this high-risk population.

## Supporting information

S1 Fig PRISMA flowchart to show process of study selection. (PDF)

S2 Fig Assessment of quality of randomized controlled trials, using the RoB2.0 tool. (TIF)

S3 Fig Funnel plot for assessment of publication bias in relation to risk of A) preterm delivery; B) low birth weight baby; C) admission to NICU; D) low APGAR score; E) foetal death; F) chorioamnionitis; G) PPROM; H) caesarean delivery. (PDF)

S4 Fig Risk of preterm birth in those undergoing ultrasound-indicated cerclage, compared to history-indicated cerclage, in studies with randomized controlled design. (TIF)

S5 Fig Comparison of mean gestational age (in weeks) among ultrasound-indicated cerclage and history-indicated cerclage, in studies with randomized controlled design. (TIF)

S1 File Retrieved studies after removing duplicates. (XLS)

S2 File Raw data and. (RAR)

S3 File PRIMSA checklist. (DOCX)
