# Supplementary material for: Comparison of pregnancy outcomes after history-indicated and ultrasound-indicated cervical cerclage: A systematic review and meta-analysis
Source: PLoS One. 2025 Aug 14;20(8):e0328564. doi: 10.1371/journal.pone.0328564 (PMC12352640; doi:10.1371/journal.pone.0328564)
Supplement: S1 Fig — (PDF) [file pone.0328564.s001.pdf]

PRISMA 2020 flow diagram for new systematic reviews which included searches of databases and registers only

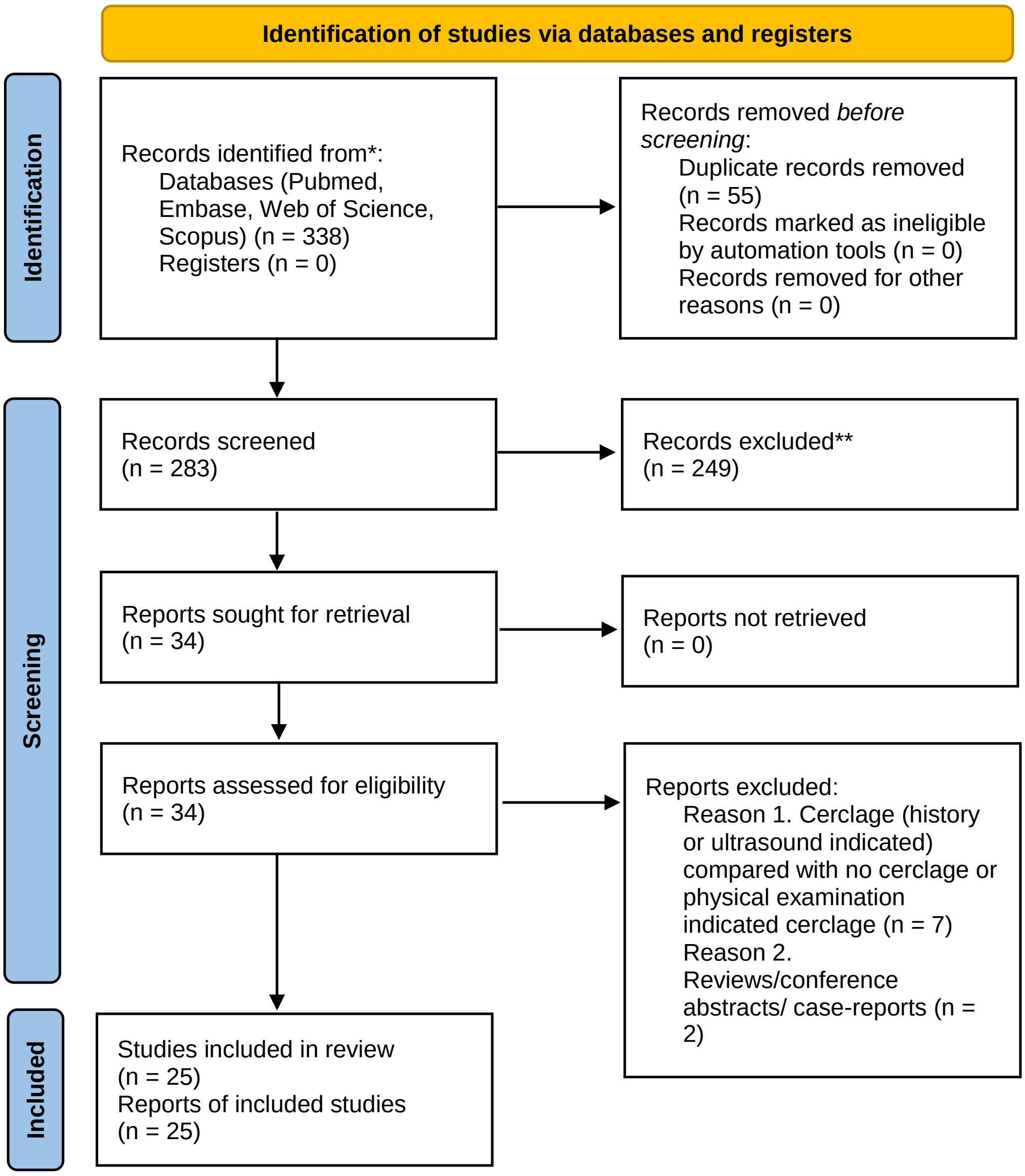

*Consider, if feasible to do so, reporting the number of records identified from each database or register searched (rather than the total number across all databases/registers).

**If automation tools were used, indicate how many records were excluded by a human and how many were excluded by automation tools.

Source: Page MJ, et al. BMJ 2021;372:n71. doi: 10.1136/bmj.n71.

This work is licensed under CC BY 4.0. To view a copy of this license, visit https://creativecommons.org/licenses/by/4.0/
