# Supplementary figures and images for: Comparison of pregnancy outcomes after history-indicated and ultrasound-indicated cervical cerclage: A systematic review and meta-analysis
Source: PLoS One. 2025 Aug 14;20(8):e0328564. doi: 10.1371/journal.pone.0328564 (PMC12352640; doi:10.1371/journal.pone.0328564)

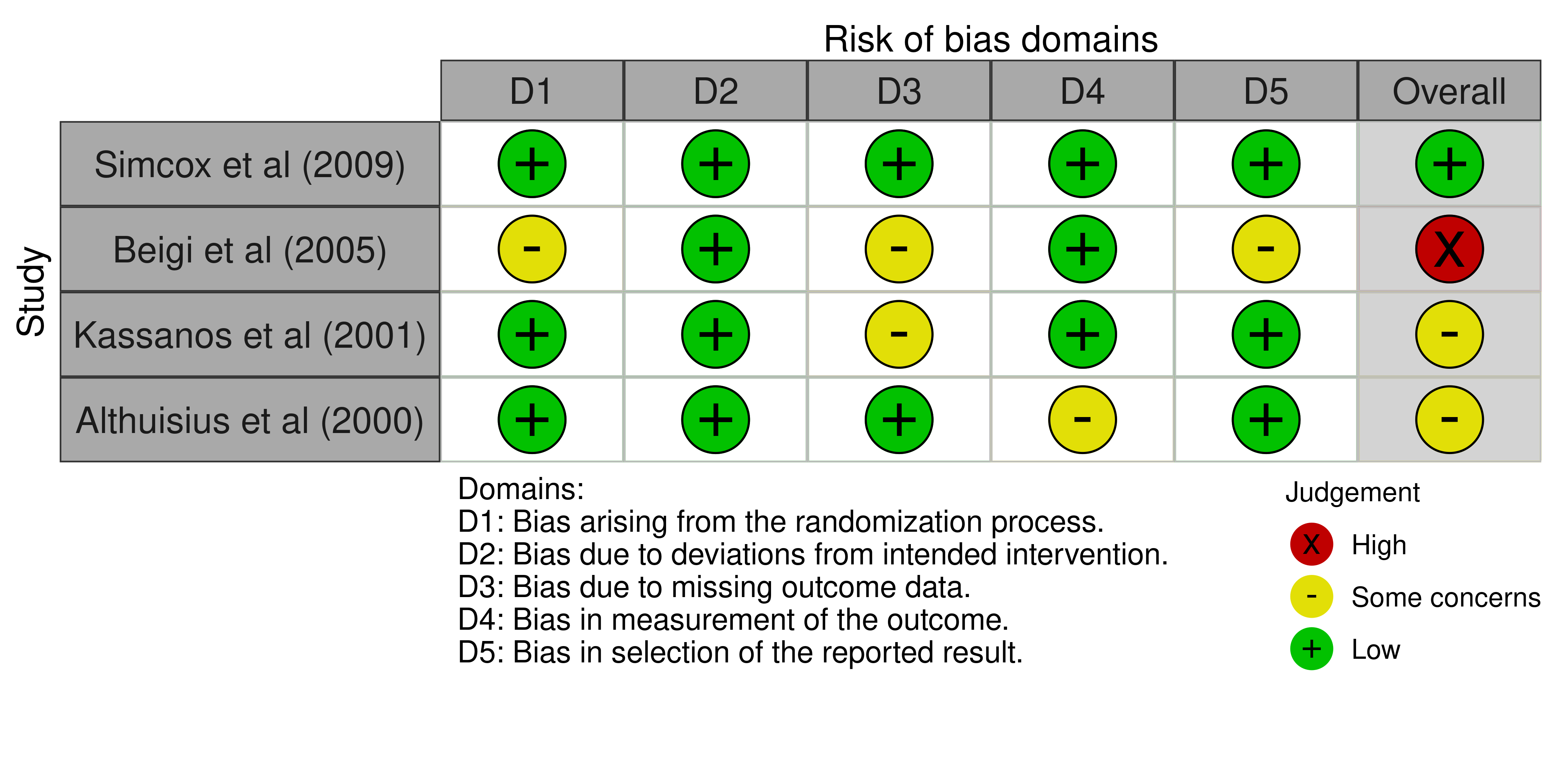

Supplement: S2 Fig — (TIF) [file pone.0328564.s002.tif]

A)

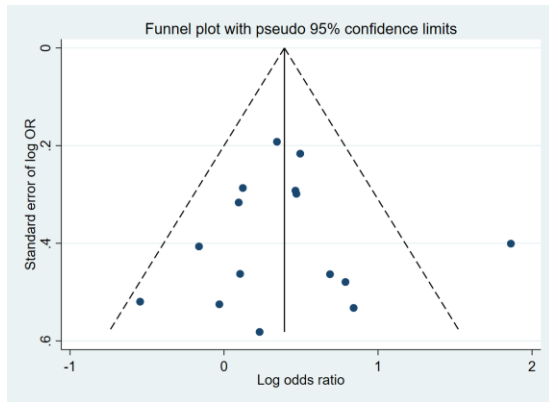

B)

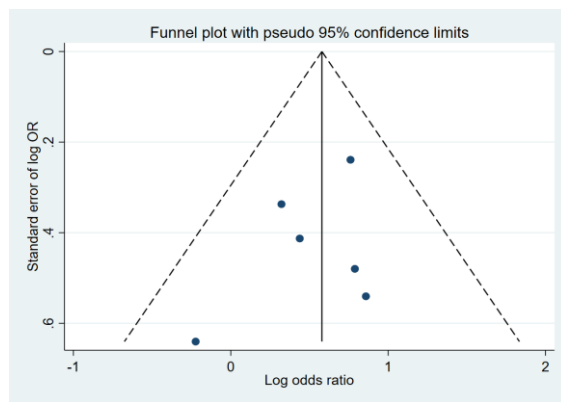

C)

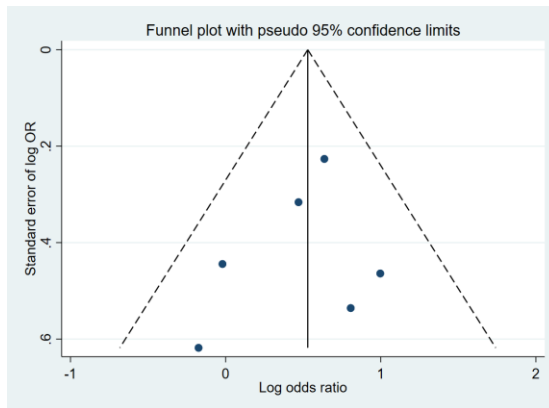

D)

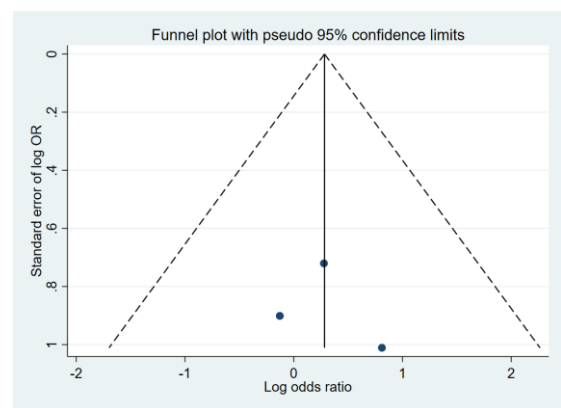

E)

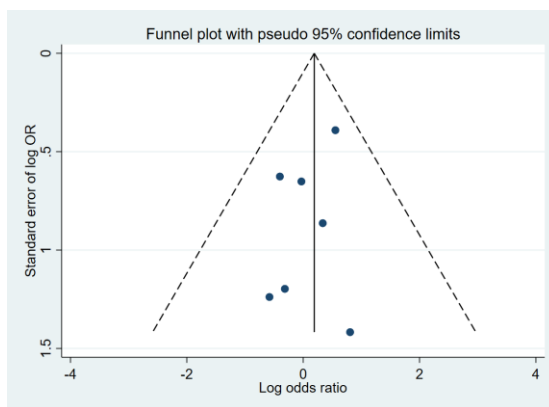

F)

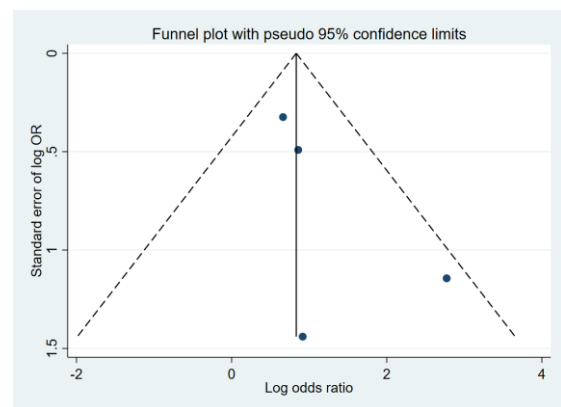

G)

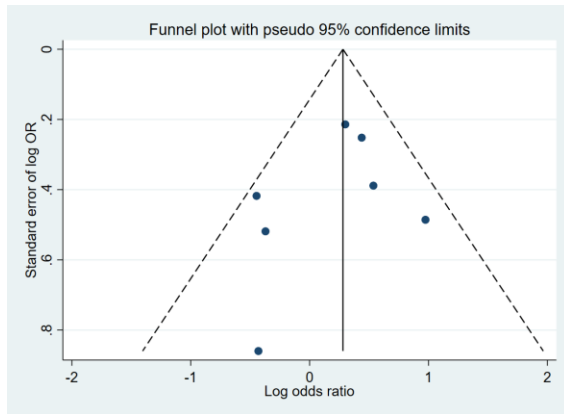

H)

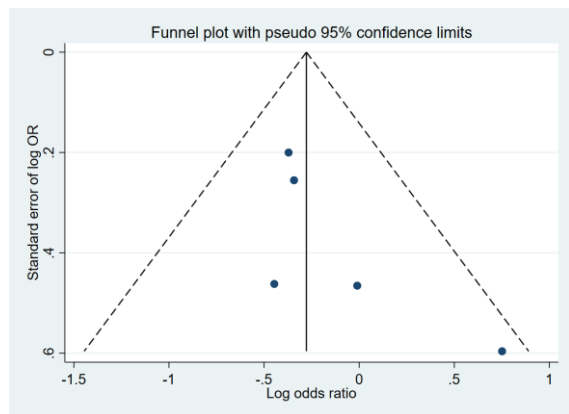

Supplement: S3 Fig — (PDF) [file pone.0328564.s003.pdf]

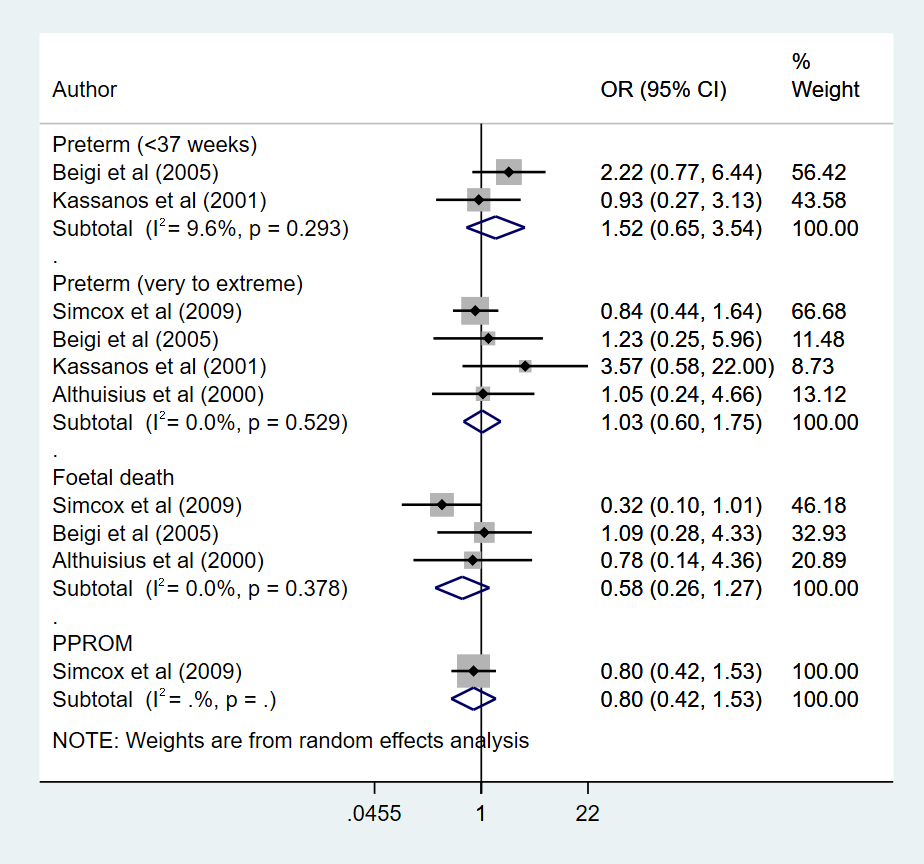

Supplement: S4 Fig — (TIF) [file pone.0328564.s004.tif]

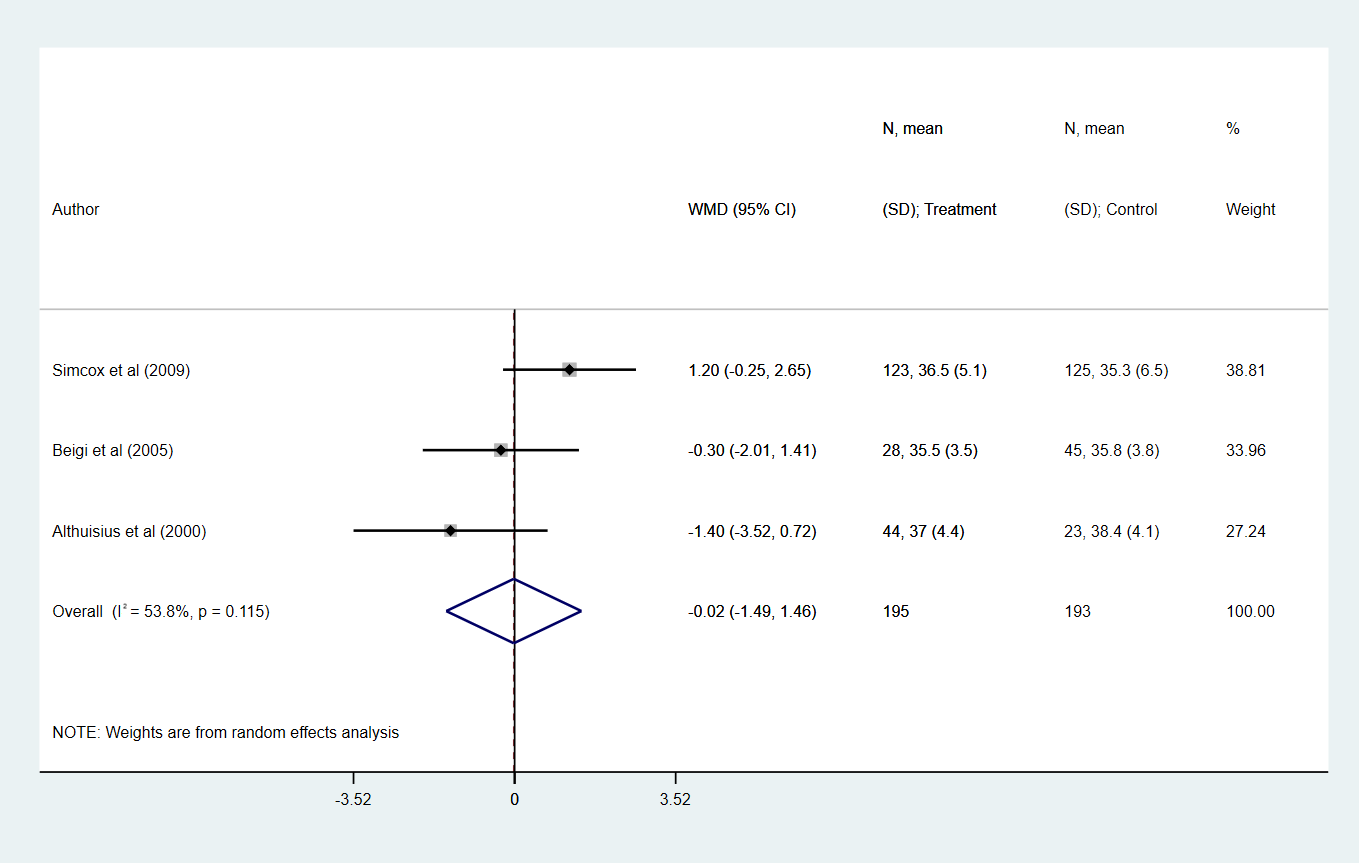

Supplement: S5 Fig — (TIF) [file pone.0328564.s005.tif]
